# Modular, rule-based modeling for the design of eukaryotic synthetic gene circuits

**DOI:** 10.1186/1752-0509-7-42

**Published:** 2013-05-27

**Authors:** Mario Andrea Marchisio, Moreno Colaiacovo, Ellis Whitehead, Jörg Stelling

**Affiliations:** 1 ETH Zurich and Swiss Institute of Bioinformatics, D-BSSE, Mattenstrasse 26, Basel 4058, Switzerland; 2 CRA-GPG Centro di ricerca per la genomica e la postgenomica animale e vegetale, Consiglio per la Ricerca e la Sperimentazione in Agricoltura, Via San Protaso 302, Fiorenzuola d’Arda (PC) 29017, Italy

**Keywords:** Composable parts, Pools of signal carriers, Gene circuit modular design, Rule-based modeling

## Abstract

**Background:**

The modular design of synthetic gene circuits via composable parts (DNA segments) and pools of signal carriers (molecules such as RNA polymerases and ribosomes) has been successfully applied to bacterial systems. However, eukaryotic cells are becoming a preferential host for new synthetic biology applications. Therefore, an accurate description of the intricate network of reactions that take place inside eukaryotic parts and pools is necessary. Rule-based modeling approaches are increasingly used to obtain compact representations of reaction networks in biological systems. However, this approach is intrinsically non-modular and not suitable *per se* for the description of composable genetic modules. In contrast, the Model Description Language (MDL) adopted by the modeling tool ProMoT is highly modular and it enables a faithful representation of biological parts and pools.

**Results:**

We developed a computational framework for the design of complex (eukaryotic) gene circuits by generating dynamic models of parts and pools via the joint usage of the BioNetGen rule-based modeling approach and MDL. The framework converts the specification of a part (or pool) structure into rules that serve as inputs for BioNetGen to calculate the part’s species and reactions. The BioNetGen output is translated into an MDL file that gives a complete description of all the reactions that take place inside the part (or pool) together with a proper interface to connect it to other modules in the circuit. In proof-of-principle applications to eukaryotic Boolean circuits with more than ten genes and more than one thousand reactions, our framework yielded proper representations of the circuits’ truth tables.

**Conclusions:**

For the model-based design of increasingly complex gene circuits, it is critical to achieve exact and systematic representations of the biological processes with minimal effort. Our computational framework provides such a detailed and intuitive way to design new and complex synthetic gene circuits.

## Background

**Figure 1:**
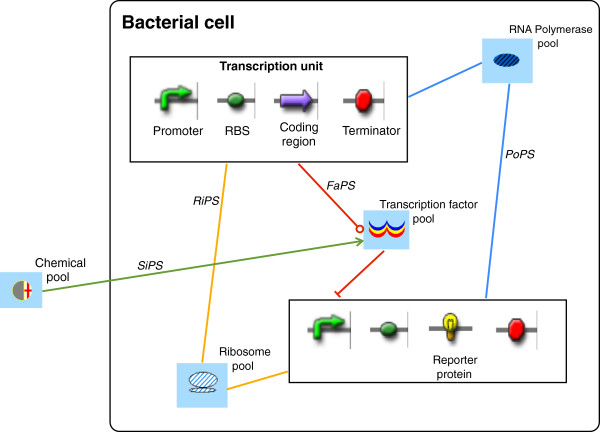
**A one-step cascade in bacteria.** This simple circuit is made of two transcription units: the upper expresses a repressor that, in the absence of chemicals, binds the promoter along the second transcription unit and suppresses the synthesis of a reporter (fluorescent) protein. When chemicals enter the cell, they bind and inactivate the repressors, switching on cell fluorescence. The role of pools as interfaces either between transcription units or between the cell and the environment is apparent. Simple straight lines here represent a mere exchange of molecules; the one ending with a circle symbolizes translation; the one with an orthogonal segment stands for repression; the one with an open arrow induction (see Additional file 1 for an outline of all the symbols used throughout this paper). FaPS means Factors Per Second and it is the flux associated with repressors from one to another transcription unit; SiPS–the flux of chemicals into the cell–stands for Signals Per Second. RNAPS–RNA Per Second–is the acronym for the flux of small RNAs, the only signal carrier not present in this circuit.

Bacterial synthetic gene circuits can be designed in an electronic fashion by wiring together Standard Biological Parts and pools of signal carriers [1]. Following the classification given by the MIT Registry (http://partsregistry.org/), Standard Biological Parts are DNA segments such as promoters, ribosome binding sites (RBS), coding regions, small RNAs, and terminators (see Figure 1). Each part is characterized by a well-defined function either in transcription or translation.

Signal carriers are molecules that play the role of b͡io-electrons: they are exchanged between parts in such a way that their fluxes (*bio-currents*) make a genetic circuit work. Originally, RNA polymerases and ribosomes were chosen as the only common signal carriers [2]. RNA polymerases bind the DNA at complementary promoters, go through RBSs and coding regions (or small RNAs), while simultaneously transcribing them into mRNA, and finally they reach a terminator and leave the DNA. Therefore, RNA polymerases scan entire transcription units and their flux (PoPS: Polymerase Per Second) has been proposed as a measure of promoter strength [3,4]. Ribosomes bind the mRNA at the RBS and translate coding regions into proteins. In analogy with RNA polymerases, their flux (RiPS: Ribosome Per Second) might be adopted to quantify the RBS strength [5].

In our representation [1,6], three more kinds of molecules act as signal carriers because of their role in transcription and translation regulation, namely transcription factors, small RNAs, and chemicals. Transcription factors are proteins that bind promoters and either prevent (repressors) or enable (activators) RNA polymerase binding. Small, antisense RNAs, on the contrary, regulate translation by binding the mRNA and forming or removing hairpin loops that are hurdles to ribosome flux. Chemicals carry out a regulatory action both a) in transcription by binding transcription factors and modifying their spatial conformation and, therefore, their activity, and b) in translation by binding and altering mRNA secondary structures such as riboswitches and ribozymes [7]. We associated a pool and a flux to each of the five signal carriers. Pools represent the cellular ‘storage’ for free molecules of signal carriers; they can be seen as *bio-batteries*, since it is their content that drives circuit activity. Furthermore, in a gene circuit design, transcription factor and small RNA pools connect transcription units, whereas chemical pools are interfaces between the whole circuit and the extra-cellular environment (see Figure 1).

Transcription and translation efficiency depend on promoter and RBS features, respectively. Transcription is modulated by various affinities: between RNA polymerases and promoter sequences; between transcription factors and their corresponding DNA binding sites (operators); between chemicals and transcription factors. Analogously, the accuracy with which both small RNAs and chemicals modify the mRNA secondary structure influences translation strength. Moreover, transcription factors and chemicals can bind DNA (the former) and mRNA (the latter) cooperatively.

In our bacterial part model, we considered only promoters and RBSs regulated by no more than two regulatory factors [1,8]. This assumption turned out to be sufficient to reproduce *in silico* most of the synthetic circuits realized in *E. coli* during the first half of the last decade [9]. However, in recent years, the emphasis has shifted to synthetic biology applications in more complex organisms and several circuits in eukaryotic cells have been engineered [10-13]. Therefore, a detailed description of *eukaryotic gene parts* is necessary to properly model and simulate *in silico* synthetic constructs for yeast and mammalian cells. Moreover, cellular compartments of various volumes, such as nucleus and cytoplasm, have to be taken explicitly into account. Since they host reactions without counterparts in prokaryotes, new pools have to be introduced.

Several other features distinguish eukaryotic from prokaryotic transcription and translation, and corresponding modular mathematical descriptions are missing to date. Once transcribed, mRNA undergoes splicing and maturation in a eukaryotic cell’s nucleus before being transported into the cytoplasm where it is translated. Moreover, eukaryotic mRNA does not have a unequivocal sequence (such as the Shine-Dalgarno one in bacteria) recognized by the ribosomes. Therefore, the RBS as a part *per se* is no longer necessary and the binding site for the ribosomes is embedded in the protein coding region. Furthermore, the nucleus has a spliceosome pool, and the cytoplasm contains as many mRNA pools as there are coding regions in the circuit. While eukaryotic riboswitches/ribozymes-mediated translation regulation does not show any particular difference to bacteria, RNA interference (RNAi) includes more steps than the sole antisense RNA base-pair binding. Indeed, siRNAs (small interfering RNAs) are processed in the nucleus and exported to the cytoplasm where they bind the so called RISC (RNA Induced Silencing Complex). In this configuration, they bind and cleave their targets on mRNA, after which the mRNA is degraded rapidly [14]. We therefore include a pool for the Dicer enzyme in the nucleus (see the modeling below for more details) and one for the RISC in the cytoplasm.

Recent advances in transcription factors engineering[15,16] and RNA-based synthetic biology [17-19] raise a further demand: the model of parts such as promoters and coding regions has to take into account an increasing number of operators and mRNA binding sites, respectively (in principle, this requirement holds also for bacterial systems). However, this means an exponential growth in the number of species and reactions for these parts. For example, in our previous model, bacterial promoters had no more than two operators. Since each operator can assume two states (free and taken by a transcription factor), only four possible configurations were possible, and for a unique transcription factor, only four binding reactions were present. If, for instance, we increase the number of operators to six, we will have 64 possible configurations and 192 binding reactions. This is what is referred to as the *combinatorial explosion* problem.

*Rule-based* modeling approaches tackle this combinatorial explosion of species and reactions. A biological system is specified by: a) a general description of the species types and their possible states (in our example: a transcription factor that can be bound or unbound and a promoter with six operators that can be either free or bound); b) a list of *seed* species, i.e., the ones present before any interaction takes place (an unbound transcription factor and a promoter with six free operators); c) a set of rules that describe the interactions between the species (unbound transcription factors bind free operators). According to these input specifications, software such as BioNetGen [20] and the Kappa Calculus [21] compute all the species and reactions of the system under consideration (see Additional file 1 for a schematic representation). These tools have been proved to be extremely efficient at giving a compact representation of biological systems. For instance, the cBNGL (compartmental BioNetGen language) has provided a detailed description of the EGF signaling cascade in mammalian cells [22].

However, rule-based languages cannot be used to generate directly models for single composable parts (and pools) that interact via the exchange of fluxes of molecules such as signal carriers, as in our model.

In contrast, the Model Description Language (MDL) [23] adopted by the software ProMoT [24] is highly modular, and all our composable parts and pools find a clear MDL representation.

**Figure 2:**
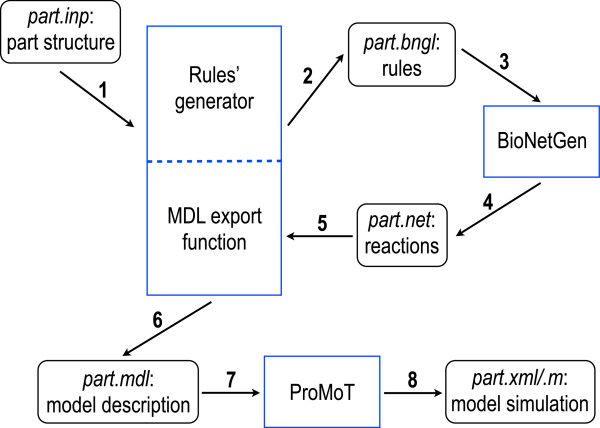
**Computational architecture for the design of modular, rule-based parts and pools.** An input (text) file is converted into an MDL file in six steps. MDL files corresponding to parts and pools are loaded into ProMoT where they are wired up into circuits. Finally, ProMoT allows exporting gene circuits into SBML and Matlab format in order to simulate their dynamics.

In this work, we present an extension of our modeling tool [1] to build *combinatorial* composable biological parts for both complex prokaryotic and eukaryotic systems via the joint usage of BNGL and MDL. Figure 2 shows a schematic of the framework (see also Methods). A high-level description of part structures (made of, e.g., binding molecules and sites) is converted into rules that serve as inputs for BioNetGen. BioNetGen elaborates a list of species and reactions that is parsed into an MDL file containing the proper interface a part needs to be connected to other parts and pools. Within ProMoT, parts and pools can be wired into circuits. The circuits can then be exported into formats suitable for simulations such as SBML [25] and Matlab (Mathworks, Nantucket / MA).

This modeling framework represents a novelty in the field of computational synthetic biology. Several other computational tools for the modular design and analysis of synthetic gene circuits are available [26-33]. They present various features such as simulation environments, connection to DNA-sequence databases, internal languages (e.g. Eugene [34], Antimony [35]), and rule-based grammars [36,37] for circuit specification and design automation. However, they have been tailored to prokaryotic systems only and none of them implements models for eukaryotic parts.

The paper is structured as follows: In the next section, we provide a detailed description of our novel models for eukaryotic parts and pools (see Methods for details). As an example application, we subsequently show how to build within this framework an *RNAi-based logic evaluator*[38] and a possible counterpart based on transcription regulation. The discussion of simulation results is accompanied by considerations about the improvements with respect to prior work and future perspectives.

## Results and discussion

### Models for eukaryotic parts and pools

With the joint usage of MDL and BNGL, we propose models for eukaryotic parts and pools that arise, when possible, from the corresponding bacterial modules [1,8]. We aim at giving part descriptions that are useful for synthetic biology applications and do not pursue an exhaustive representation of all the possible interactions that govern transcription and translation in eukaryotes. Moreover, not all the mechanisms behind mRNA and protein synthesis are well known, and the values of several kinetics parameters have not been measured yet. Therefore, despite the power of a rule-based modeling approach, one has to find a proper trade-off between model granularity and available knowledge in order to obtain a meaningful model that can predict synthetic gene circuit dynamics and performance. Specifically, among the set of composable parts and pools, only promoters and coding regions require a rule-based modeling approach because of their potentially complex structure where several binding sites for regulatory factors are present together with either an RNA polymerase or ribosome binding site, respectively.

A more exhaustive description of eukaryotic systems might take into account mechanisms that have been neglected here. For instance, cell metabolic reactions can be described by a network of pools that either store free molecules (e.g. kinases and phosphatases) or that represent enzymatic reactions (e.g. phosphorylation and dephosphorylation). Furthermore, part models presented below might be enriched by considering also operator positional effects and transcription squelching, for instance. Such a precise picture might be useful for the analysis of specific cellular phenomena–and the evaluation of the corresponding kinetic parameter values–on rather simple gene circuits.

#### Promoters

To model promoters, operator position is not explicitly taken into account, but activator binding sites are supposed to be placed upstream of the TATA box whereas repressors bind the DNA between the TATA box and the TSS (Transcription Starting Site). We take a prokaryotic repression model based on competition between repressors and RNA polymerases, where DNA-bound repressors prevent RNA polymerases from reaching their binding sites and initiating transcription. The use of bacterial transcription factors in eukaryotic cells is a way of combining *orthogonal* systems that is broadly exploited in synthetic biology [39,40].

**Figure 3:**
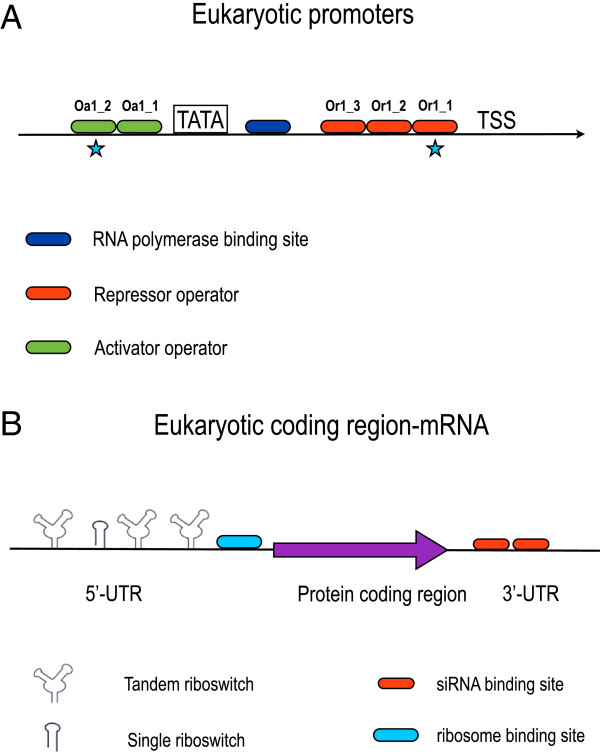
**Synthetic eukaryotic promoter and mRNA.A**) In the configuration here shown, a promoter is bound by a repressor *R*_1_ and an activator *A*_1_. Every operator is labeled with the name of the corresponding transcription factor and the position with respect to the TSS (the lower the integer, the closer the operator to the TSS). A star marks the operators with the highest affinity in case of cooperativity. **B**) mRNA with four riboswitches along the 5’-UTR (three of them are tandem ones) and two siRNA binding sites on the 3’-UTR region. The ribosome binding site is sequestered by the riboswitches nearby when they are in their inactive configuration.

Different transcription factors can bind a promoter, each on *N* operators, in principle. Transcription factors of the same species can bind cooperatively. Here, as in our previous work [1], we assume that the affinity between DNA and transcription factors varies with the relative position of the operators with respect to the TSS. As for repressors, the strongest operator is the one closest to the TSS. In contrast, activators bind with higher affinity to the one furthest from the TSS and the TATA box (see Figure 3A). The binding of a transcription factor to an operator causes a rotation of the DNA such that the binding rate constant of the adjacent operator is increased [41].

RNA polymerase binds the DNA in the absence of any repressors. If it is recruited by activators, two scenarios are possible: a) if the activators do not bind cooperatively, only one of their operators has to be occupied in order to let RNA polymerase bind; b) if the activators bind cooperatively, all their operators must be bound to get transcription started. Chemicals can bind and inactivate transcription factors anchored to their operators. Depending on the presence or absence of cooperativity, the binding of a co-repressor [42] to an activator can have a different repercussion on RNA polymerase bound to the DNA. Without cooperativity, all the activator’s operators must be free to let polymerase leave the double chain. In contrast, in case of cooperativity it is enough to free the rightmost operator to destabilize the polymerase-DNA bond (see Additional file 1 for figures illustrating these interactions).

Promoter leakage is proportional to all the configurations where at least one repressor is bound or, in the absence of repressors, where there is no activator whatsoever on the DNA (without cooperativity) or all the right-most operators (with cooperativity) are free.

#### Coding region, siRNA and mRNA pools, terminators

As already mentioned above, eukaryotic cells do not have an RBS. Therefore, translation regulation–together with gene expression–concerns the coding region. Here, in contrast to our bacterial framework, each coding region has a corresponding mRNA pool in the cytoplasm. mRNA pools are connected to the ribosome pool and, potentially, to chemical and siRNA pools as well. In the nucleus, mRNA is transcribed and spliced, and it becomes mature inside the coding region part. Free molecules of the spliceosome have their own pool and they interact with the immature mRNA by following a Michaelis-Menten (enzyme-like) scheme. All the other steps of mRNA maturation and transport into the cytoplasm are lumped into a single reaction to minimize the model’s number of kinetic parameters.

Translation regulation occurs either via riboswitch activation/deactivation or RNA interference. As in the promoter case, position along the mRNA is not explicitly taken into account. However, riboswitches are normally placed on the 5’-UTR (Untranslated Region) [43] whereas siRNA binding sites lie on the 3’-UTR [44] (see Figure 3B).

Riboswitches are, essentially, RNA hairpin loops that can prevent ribosome binding. In our framework, they assume two different states: active (*on*) and inactive (*off*). Only the active state allow ribosome binding to the mRNA. Riboswitches change their state upon chemical binding to their *aptamers*. Only when all the riboswitches’ aptamers are *on*, ribosomes are allowed to bind the mRNA and to start translation. As an improvement of our previous representation [8], here we explicitly consider single as well as tandem riboswitches with one or two aptamers, respectively. Tandem riboswitches can be bound by a unique chemical species or by two different species. Since homo- and hetero-cooperativity have been reported in literature [45,46], both have been taken into account in our model. In principle, *N* different riboswitches can be placed along the 5’-UTR.

RNAi interference is a regulation mechanism typical of higher eukaryotes such as mammals, but it has also been engineered into budding yeast [47]. In our framework, we suppose that a siRNA-coding region drives the formation of double-stranded small interfering RNAs in the nucleus. They undergo a splicing operation after interacting with the Dicer enzyme and are then exported to the cytoplasm as a single strand. As in the mRNA case, all the nuclear maturation processes and transport are lumped into a single reaction. Free Dicer molecules (from a distinct pool) act on double stranded RNAs following a Michaelis-Mentes scheme (analogous to the mRNA-spliceosome interaction above). In the cytoplasm, siRNA pools are connected both to the mRNA and the RISC pools. Despite its complex structure, RISC is here treated as a single molecule that binds an siRNA in the siRNA pool and brings it to its target mRNA. Once the siRNA is bound to the mRNA, the mRNA is cleaved and rapidly degraded, and any ribosome along the mRNA is released. Each siRNA can bind to any of *N* different sites placed on the mRNA’s 3’-UTR (see Additional file 1).

mRNA half life strongly influences the dynamics of synthetic gene circuits. Terminators introduce loop structures at the end of the mRNA sequence which may considerably alter the mRNA’s stability [48]. Therefore, in contrast to bacteria, eukaryotic terminators are characterized by specifying the decay rate of the mRNA (or siRNA) produced by the transcription unit they belong to.

Transcription factors and fluorescent proteins are synthesized inside the cytoplasm. The former are imported into the nucleus where they exert their regulatory action on the DNA, and the latter flow into a pool placed in the cytoplasm, since they are not normally localized into the nucleus.

**Figure 4:**
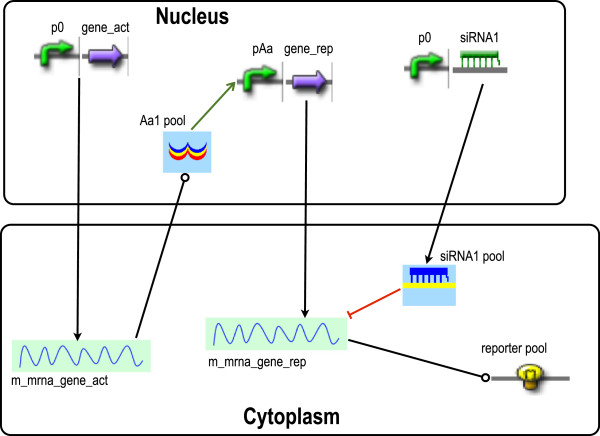
**Gene circuits in eukaryotic cells.** In the circuit, fluorescence expression is under the control of an activator and an siRNA. For the sake of simplicity we do not show all the terminators and all pools; RNA polymerase, ribosome, spliceosome, Dicer, and RISC pools were removed. Every full arrow represents a transcription process.

In Figure 4 we provide a graphical representation of a simple gene circuit made of parts and pools in a eukaryotic cell. A more detailed model description is available in Additional file 1, including all the circuit reactions and rules in BNGL.

### Application: logic evaluator in mammalian cells

As a benchmark for our eukaryotic part and pool model, we chose the RNAi logic evaluator by Rinaudo *et al.*[38]. In this work, Boolean gates of varying complexity have been implemented via siRNA-dependent translation regulation; siRNA expression is under the control of endogenous signals. In our previous work on the automatic design of gene digital circuits [8], we presented alternative solutions–in bacteria–to the circuit associated with the Boolean function: $\left(a\;\wedge \;b\;\wedge \;d)\;\vee \;(\bar{a}\;\wedge \;c\right)$, where $\bar{a}$ stands for *NOT*(*a*), ∧ for *AND*, and ∨ for *OR*. As inputs, we considered four external chemicals that interact with promoters and RBSs. However, since our composable parts accommodated at most two binding sites on DNA or mRNA, we could not predict a circuit design that only employs transcription or translation control, respectively. With the new set of eukaryotic parts and pools, we are now able to reconstruct an RNAi-based logic evaluator that is close to the original one, and to design an alternative circuit that performs the same Boolean function via transcription regulation alone.

**Figure 5:**
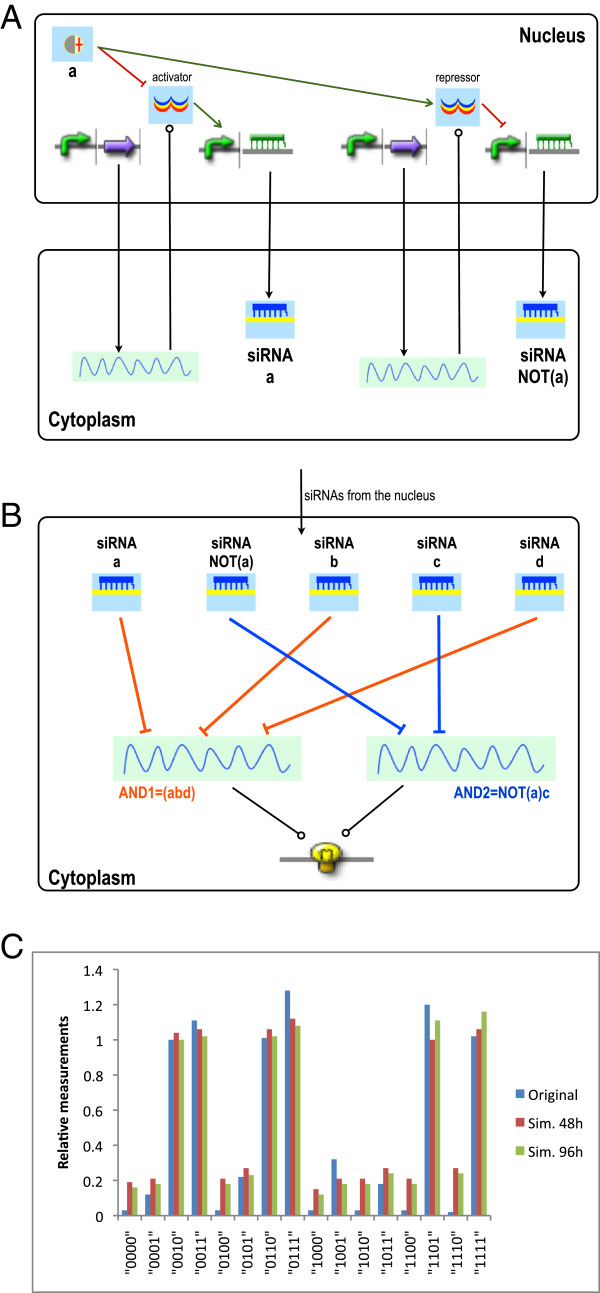
**RNAi-based logic evaluator.A**) Conversion of a chemical into a siRNA. Following Rinaudo *et at.*, signal *a* inhibits siRNA-*a* and promotes siRNA-$\bar{a}$ expression. This double function is mimicked by requiring that this chemical binds and deactivates two different transcription factors. When *a* is present, only siRNA-$\bar{a}$ is transcribed thus–neglecting other signals in the circuit– *AND*_2_ mRNA is cleaved whereas *AND*_1_ produces fluorescence; vice versa in absence of *a*. **B**) Cytoplasmic *AND* gates. **C**) Comparison of *in silico* simulations and *in vivo* measurements. For each truth table entry, we calculated the ratio between the corresponding fluorescent protein concentration and the minimal 1-output value (absolute values are shown in Additional file 1). This is the procedure followed by Rinaudo and co-authors (in their case, the lowest 1 concentration is at the entry “0010”). They, however, measured fluorescence.

In the Rinaudo *et al.* version of the circuit, each Boolean variable corresponds to an endogenous signal. When a signal is present, its corresponding siRNA is “inactivated” that is, no longer produced. Moreover, signal *a* also activates the siRNA associated with the $\bar{a}$ variable. However, siRNA synthesis is not shown explicitly. Therefore, in our circuit we decided to put siRNA-*a*,-*b*,-*c*, and -*d* under the control of an activator that is inhibited when bound by the corresponding input chemical, whereas the transcription of siRNA-$\bar{a}$ is controlled by a repressor that is also inhibited by signal *a* (see Figure 5A). Both *AND* gates are transcription units that produce the same fluorescent protein, the circuit output. Each siRNA has two binding sites on its target mRNAs, so the *AND*_1_ gate (*a* ∧ *b* ∧ *d*) has a total of 6 binding sites, whereas 4 lie on *AND*_2_$\left(\bar{a}\;\wedge \;c\right)$–see Figure 5B. To quantify the complexity of this circuit: each mRNA pool where either an activator or a repressor is transcribed has 5 internal species, 9 reactions, and 2 exchange fluxes; the mRNA pool associated with the *AND*_1_ (*AND*_2_) gate has 17 (13) species, 45 (33) reactions, and 5 (4) exchange fluxes. Overall, the circuit contains 197 species and 474 reactions. We assume that parts of the same type–such as promoters regulated by an activator or siRNA coding regions–are identical. This means that we have to specify only a limited amount of parameter values with respect to the number of circuit reactions (see Additional file 1 for details).

As shown in Figure 5C, deterministic circuit simulations correctly reproduce the circuit’s truth table in terms of high/low reporter outputs, but also with respect to the quantitative outputs, without specific tuning of model parameters. The choice of deterministic simulations is justified by the fact that both logic evaluators exceed 100 proteins in signal separation (see Additonal file 1), which we shown to be a condition for large Boolean networks to be insensitive to stochastic noise [8]. In our simulations, we first let the circuit get to the steady state in the absence of chemicals (96 hours). Then we fed it with the inputs in order to calculate all 16 entries of the truth table. After 48 hours (i.e, the time considered in the original work), a clear signal separation is already reached. The separation does not improve substantially if we simulate the circuit for 96 hours. Discrepancies with the published measurements mainly concern the logical 0 levels. These probably reflect the fact that our circuit is not identical to the reference circuit since we had to choose arbitrarily how to design the siRNAs’ expression. Moreover, our knowledge of RNAi kinetic parameter values is still quite limited and adaptations of parameter values or the implementation of more detailed models for RNAi [49] might further improve our results. In this paper, however, we want to show that our framework based on composable parts and pools generated via a rule-based modeling approach is applicable to the design and analysis of eukaryotic cells, and a more detailed analysis of the parameter space of such systems is left to a future work.

**Figure 6:**
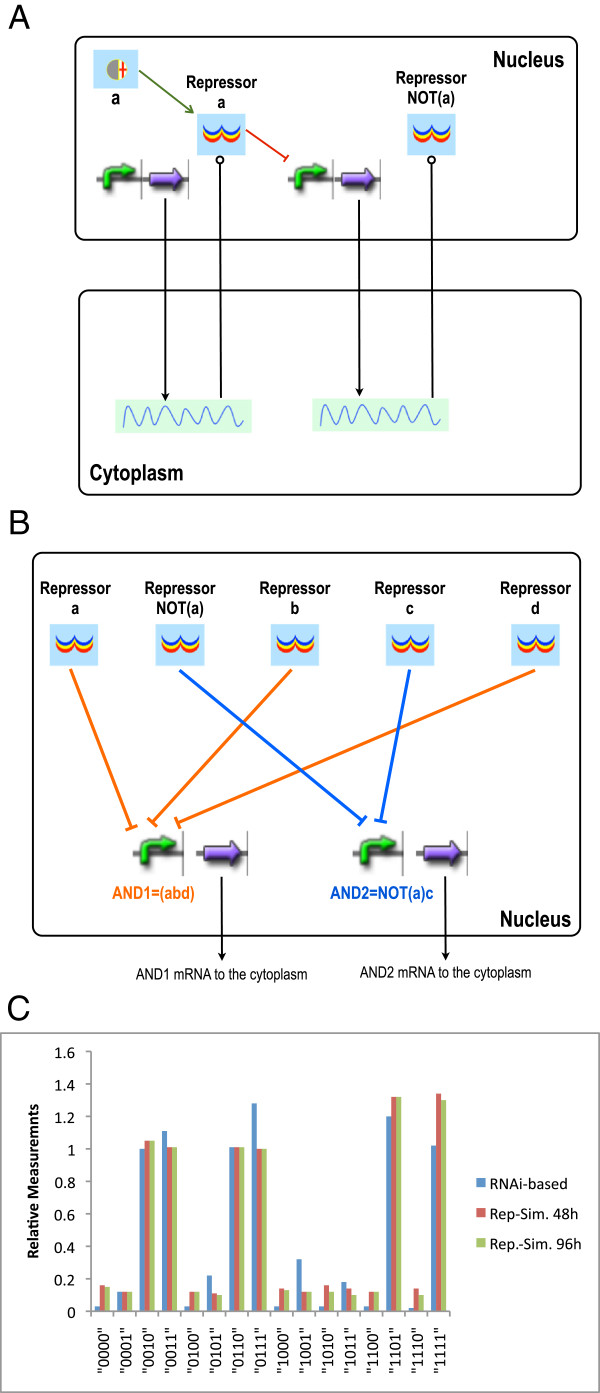
**Transcription repression-based logic evaluator.A**) Conversion of a chemical into a repressor. When signal *a* is present, only repressor $\bar{a}$ is expressed. Therefore–neglecting the other signals– *p*_*and*1_ is not regulated and can lead to fluorescence production; vice versa when *a* is absent. This configuration requires two genes less than the siRNA-based one. **B**) Nuclear *AND* gates. **C**) Comparison of *in silico* simulations and *in vivo* measurements. Calculations are performed as in the RNAi-based circuit.

In the transcriptional version of this circuit, siRNAs are replaced by repressors (see Figure 6A-B). Every repressor binds non-cooperatively to two operators. Therefore, symmetrically to the original circuit, *AND*_1_ is a transcription unit whose promoter (*p*_*and*1_) is regulated by three repressors and it contains a total of 6 operators; *AND*_2_’s promoter (*p*_*and*2_) is controlled by two repressors and hosts 4 operators. These promoter configurations show a high degree of complexity: *p*_*and*1_ hosts 65 species, exchanges 12 fluxes, and contains 834 reactions; *p*_*and*2_ hosts 17 species, exchanges 7 fluxes, and contains 130 reactions. The promoter that leads to the synthesis of the repressor associated with $\bar{a}$ has a configuration (2 operators) close to the most complex one we could achieve with our old set of bacterial parts: 5 species, 6 fluxes, and only 22 reactions. Although *p*_*and*1_ and *p*_*and*2_ have the same number of binding sites as the mRNAs for *AND*_1_ and *AND*_2_ in the RNAi-based circuit version, the promoters’ species are much more numerous than the corresponding mRNAs’, because mRNA is degraded as soon as one siRNA binds (states where more than one siRNA is bound are forbidden). Overall, the transcription-based version of the circuit is made of 187 species and 1165 reactions. Hence, our modular, rule-based modeling approach turns out to be extremely useful in designing systems with complex promoters.

The transcriptional version of the logic evaluator also reproduces the circuit truth table faithfully (see Figure 6C). The final 1 and 0 output levels (concentration of the reporter proteins) are higher than the ones in the RNAi-based version of the circuit because more mRNA is transcribed and more proteins are expressed when all the regulations occur at the DNA level (see Additional file 1).

## Conclusions

We have presented a new version of our computational framework based on composable parts and pools for the design of synthetic gene circuits. As a novelty, we combined a rule-based modeling approach–via the BioNetGen Language–with the modular design of biological systems–through the MDL coding. This method allows for the construction of interconnectable genetic modules with high numbers of species and reactions such as promoters, bacterial RBS, and eukaryotic mRNA pools. We provided evidence for the validity of our approach by designing and simulating complex eukaryotic Boolean circuits such as the RNAi-based logic evaluator [38] and its alternative configuration based on promoter regulation only. With both circuits we were able to reproduce the truth table faithfully.

At present, part and pool models are based on full mass-action kinetics. However, since several parameter values are still not known, we plan to perform a detailed investigation of the parameter space of mechanisms such as RNA interference and mRNA splicing. Moreover, in some cases reactions could be lumped into Hill function-based kinetics (already supported both by BioNetGen and ProMoT) in order to simplify some interaction schemes and to reduce the number of reactions and unknown parameters in the system.

A possible tool extension might exploit a new, recently developed ProMoT feature: the Process Interaction Model [50] (PIM) concept that gives a compact specification of a rule-based model and has been applied to the modeling of signaling pathways. This could lead to a fully rule-based, modular design of synthetic signaling networks, from the receptor membrane proteins down to the genes regulated in the nucleus.

Finally, our framework is highly abstract and still misses a connection with real DNA sequences: for this reason, a future link to the Synthetic Biology Open Language (SBOL) [51,52] is under study.

## Methods

### Modular, rule-based modeling design

As stated above, only promoters, bacterial RBSs, and eukaryotic coding regions (or, to be more precise, the pools of their corresponding mRNA) require a rule-based modeling approach. Part structure is specified into an input file. Here, the binding site number, the kind of regulatory factors acting on the part, their mutual interactions (i.e. cooperative or not), and their activation or inhibition via chemicals have to be specified. Moreover, a set of kinetics parameters is required too. Our program converts this information into rules that represent generic descriptions of the reactions that take place in the part. Kinetic parameters, molecules, *seed* species (i.e. the molecules present at the beginning of the computation together with their initial state and concentration), and reaction rules are written to an BNGL file (part.bngl). BioNetGen then reads the part.bngl file, calculates all the species and reactions involved in the part, and writes them to the part.net file. At this point, our program takes part.net as an input and converts its content into an MDL file (part.mdl).

In our previous software version [1], MDL files for parts and pools contained ODE systems. Here, we adopted a new representation [53] where ODEs are substituted by reactions, species (called *storage-intras*), and *adapter-fluxes*, i.e., entities that handle the exchange of fluxes of molecules with external modules and permit to build the part interface. Indeed, adapter-fluxes have two terminals: one is connected to either an internal storage-intra or a reaction, the other to a part terminal. The export function from part.net to the MDL format converts species into *storage-intras*–if they belong to the part–or into adapter-fluxes–if they belong to a pool, such as RNA polymerase and ribosomes, or they are *fictitious* (such as *Pol*^*cl*^, RNA polymerases in the promoter clearing phase, see [54] for a detailed explanation). Moreover, a library of all the reactions present inside the parts is created such that the part.mdl file can be loaded into ProMoT. Finally, links among storage-intras, adapter-fluxes, and reactions are automatically computed: they follow in a straightforward way from the definition of reaction products and educts. Figure 2 provides a schematic representation of this computational architecture.

### Software implementation

Our software is a set of Perl and Python scripts. Scripts for promoters, bacterial RBSs, and eukaryotic coding regions call BioNetGen to compute all the part or pool reactions and species. Eukaryotic coding region and siRNA scripts generate two MDL files: one for the nuclear part and the other for the corresponding mRNA/siRNA pool in the cytoplasm. Once written, part and pool MDL files can be loaded into ProMoT and the cellular nucleus and cytoplasm designed, separately, in a drag-and-drop way. Both the nucleus and the cytoplasm have to be saved as *modules*. Afterwards, they are converted into *compartments* (a different object class in ProMoT) via the python script “compartment_parser.py”. Finally, by running another python script, “link_compartment.py”, the two compartments are connected to form a cell that, in ProMoT, is an instance of the class *sbml-model*. All the circuit components (parts, pools, and corresponding reactions) are written in a single MDL file that can be loaded into ProMoT to visualize, modify if necessary, and finally export the system to SBML or Matlab format. All the simulations presented in this paper have been performed with COPASI [55]. The software is available on request from the authors.

## Competing interests

The authors declare that they do not have competing interests.

## Authors’ contributions

Main project’s idea: MAM, JS. Modeling: MAM, MC, EW. Simulations and data analysis: MAM. Paper writing: MAM, MC, EW, JS. All authors read and approved the final manuscript.

## Supplementary Material

Additional file 1 **Supplementary material.** Supplementary Material contains the list of parameter values we used for the simulations of the two logic evaluators and the simpler circuit where a reporter protein is regulated by an activator and an siRNA. This small circuit is described in details: for each of its parts and pools we give all the reactions, the corresponding BNGL rules, and the parameter values that have to be specified as inputs. Results from the simulations of both kinds of circuits are reported. Moreover, figures that elucidate some interactions (at DNA and mRNA level) considered in our framework have been inserted. Click here for file
