# Supplementary material for: Modular, rule-based modeling for the design of eukaryotic synthetic gene circuits
Source: BMC Syst Biol. 2013 May 27;7:42. doi: 10.1186/1752-0509-7-42 (PMC3680069; doi:10.1186/1752-0509-7-42)
Supplement: Additional file 1 — Supplementary material. Supplementary Material contains the list of parameter values we used for the simulations of the two logic evaluators and the simpler circuit where a reporter protein is regulated by an activator and an siRNA. This small circuit is described in details: for each of its parts and pools we give all the reactions, the corresponding BNGL rules, and the parameter values that have to be specified as inputs. Results from the simulations of both kinds of circuits are reported. Moreover, figures that elucidate some interactions (at DNA and mRNA level) considered in our framework have been inserted. [file 1752-0509-7-42-S1.pdf]

Modular, rule-based modeling approach to the design of eukaryotic synthetic gene circuits. Supplementary Material

M.A. Marchisio. M. Colaiacovo, E. Whitehead, and J. Stelling

Contents

1 Mass-action kinetics **2**

2 Eukaryotic parts and pool: parameter values **3**

3 Interactions on DNA and mRNA **6**

4 Eukaryotic gene circuits: an example **9**

4.1 The constitutive p0 promoter . . . 9
4.2 The regulated pAa promoter . . . 10
4.3 Coding region for proteins: gene_aa1 and gene_rep . . . 12
4.4 Coding region for siRNAs: siRNA1 . . . 13
4.5 Terminators . . . 14
4.6 The activator pool (Aa1 pool) . . . 14
4.7 m_mrna_gene_act . . . 14
4.8 m_mrna_gene_rep . . . 16
4.9 The siRNA pool (siRNA1 pool) . . . 18
4.10 The reporter protein pool . . . 18
4.11 Other pools . . . 19
4.12 Simulations . . . 20

5 Logic evaluator simulations **21**

Chapter 1

Mass-action kinetics

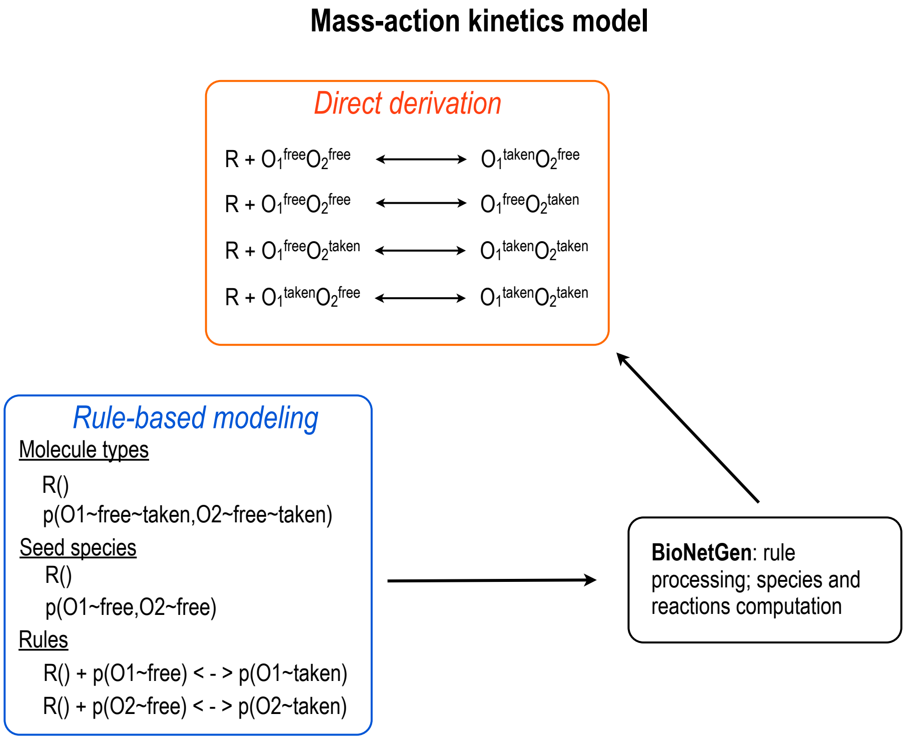

Figure 1.1: **Modeling with mass-action kinetics.** In a simple system, repressor R binds a two-operator-containing promoter. The corresponding model based on full mass-action kinetics can be easily derived by hand and contains 5 species and 4 reversible reactions. Rule-based modeling approach requires a more abstract picture of the system. This is given by: 1) the types of molecules present in the system and the states they exhibit; 2) the species present at the beginning of the computation (seed species: an unbound repressor and a promoters whose operators are free; 3) rules that describe the interactions between the species (a repressor binds a free operator no matter the state of the other operator). This information is passed as an input to programs such as BioNetGen that compute all the system's species and reactions returning a complete full mass-action kinetics model.

Chapter 2

Eukaryotic parts and pool: parameter values

The following parameter values have been used in the simulations of the logic evaluators and the simple eukaryotic circuit discussed in the next chapters and in the main text.

VOLUME	Value	Reference
$v_{nucleus}$	$3.74 \cdot 10^{-13} l$ ($374 \mu m^3$)	[8]
$v_{cytoplasm}$	$9.7 \cdot 10^{-13} l$ ($970 \mu m^3$)	[6]

POLYMERASE AND OTHER POOLS	Value	Reference
$molecules^{free}$	30000	arbitrary

PROMOTERS	Value	Reference
k_1	$10^5 M^{-1} s^{-1}$	polymerase binding rate constant[7]
k_{-1}	$1 s^{-1}$	polymerase unbinding rate [7]
k_2	$0.5 s^{-1}$	transcription initiation rate [7]
α_r	$10^9 M^{-1} s^{-1}$	repressor binding rate constant[7]
β_r	$10 s^{-1}$	repressor unbinding ratel [7]
α_a	$10^9 M^{-1} s^{-1}$	activator binding rate constant[7]
β_a	$10 s^{-1}$	activator unbinding rate [7]

CODING REGIONS (Proteins)	Value	Reference
k_{fd}	$2 \cdot 10^{-3} s^{-1}$	fast decay rate[2]
k_{1r}	$10^6 M^{-1} s^{-1}$	ribosome binding rate constant [7]
k_{-1r}	$0.01 s^{-1}$	ribosome unbinding rate [7]
k_{2r}	$0.02 s^{-1}$	translation initiation rate [7]
v_{pol}	$23.3 nt/s$	polymerase speed [11]
v_r	$24 nt/s$	ribosome speed [10]
θ_s	$10^7 M^{-1} s^{-1}$	siRNA binding rate constant; tuned
ξ_s	$0.01 s^{-1}$	siRNA unbinding rate; tuned
k_{1y}	$1500 M^{-1} s^{-1}$	spliceosome binding rate constant; tuned
k_{-1y}	$0.0017 s^{-1}$	spliceosome unbinding rate; tuned
k_{2y}	$0.033 s^{-1}$	splicing rate; tuned
k_m	$0.00055 s^{-1}$ (30min)	mRNA maturation rate [3]
ζ_r	$0.5 s^{-1}$	protein synthesis rate [7]
k_{tr}	$8.3 \cdot 10^{-3} s^{-1}$ (2 min)	nuclear import rate [1]

TERMINATORS	Value	Reference
k_d	$3.8 \cdot 10^{-5} s^{-1}$ (5h)	mRNA/siRNA decay rate, tuned from [9]
ζ	$31.25 s^{-1}$	protein unbinding rate [7]

SIRNAs	Value	Reference
k_{1d}	$1500 M^{-1} s^{-1}$	Dicer binding rate constant; tuned-like the spliceosome
k_{-1d}	$0.0017 s^{-1}$	Dicer unbinding rate; tuned-like the spliceosome
k_{2d}	$0.033 s^{-1}$	splicing rate; tuned-like the spliceosome

TF POOLS	Value	Reference
k_d	$2.8 \cdot 10^{-5} s^{-1}$ $(6.9h)$	decay rate [4]
δ	$10^9 M^{-1} s^{-1}$	dimerization rate constant [7]
ϵ	$10 s^{-1}$	dimer separation rate [7]
λ	$1.0^6 M^{-1} s^{-1}$	chemical binding rate constantl [7]
μ	$10^{-3} s^{-1}$	chemical unbinding rate [7]

RISC	Value	Reference
k_{1risc}	$3 \cdot 10^7 M^{-1} s^{-1}$	RISC binding rate constant; tuned from[2]
k_{-1risc}	$0.017 s^{-1}$	RISC unbinding rate[2]

SIGNALS	Value	Reference
γ	$10^6 M^{-1} s^{-1}$	chemical binding to tr. factors rate constant [7]
s^{free}	$0/0.01M$	initial concentrations; tuned

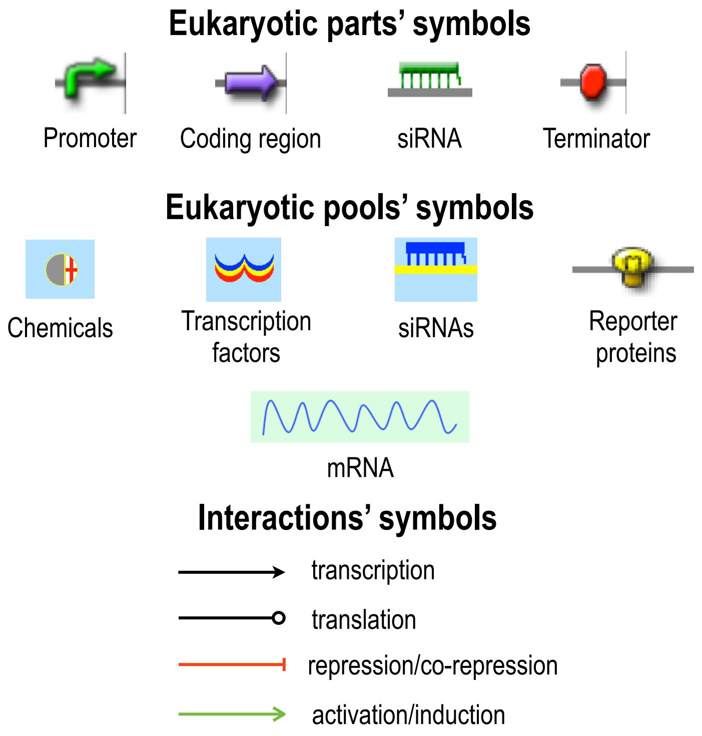

Figure 2.1: **Symbols.** Symbols used throughout the main text and the Supplementary Material.

Chapter 3

Interactions on DNA and mRNA

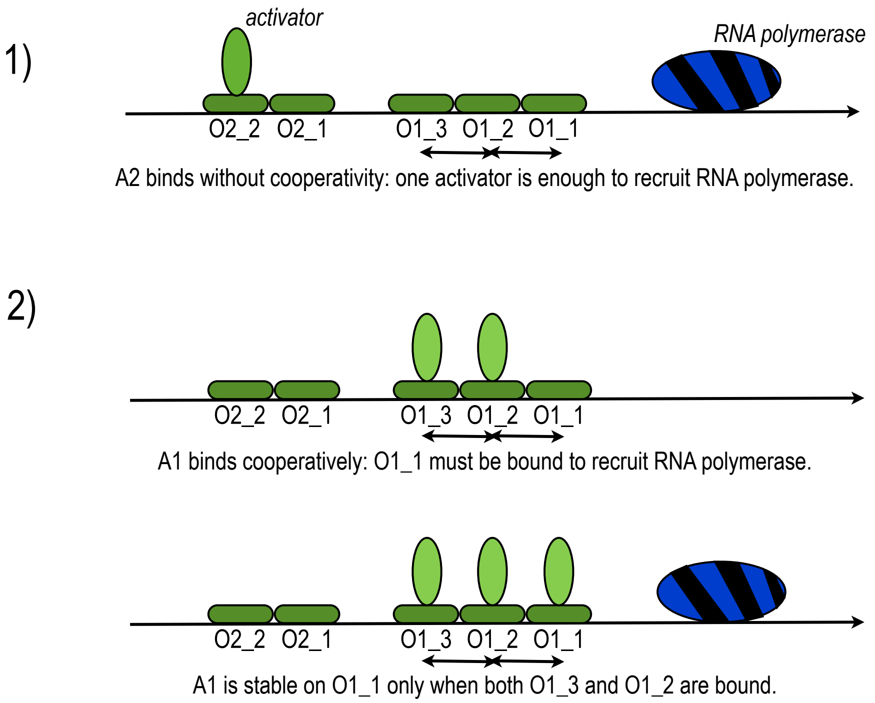

Figure 3.1: **Interaction between activators and RNA polymerases.** With this promoter configuration, RNA polymerases is recruited by two activators, A_1 and A_2. The former binds cooperatively to three operators, the latter uncooperatively to two operators. Note that the subscript a to identify the operators as activator-binding ones is here omitted. RNA polymerases binds the DNA if 1) at least one A_2 operator is bound; 2) all the three A_1 operators are taken. .

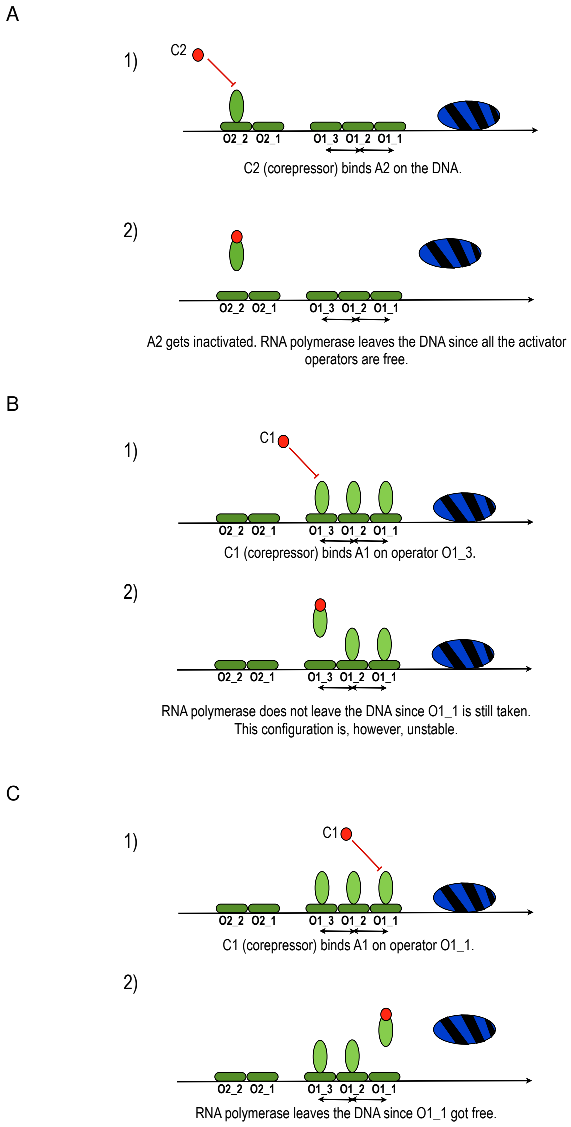

Figure 3.2: **Interaction between corepressors, activators and RNA polymerases.** A) If corepressors inactivate all the activators bound to the DNA, RNA polymerase gets free. B-C) If no A_2 is bound to the DNA, it is enough to inactivate A_1 bound to $O1_1$ to force polymerase to leave the DNA.

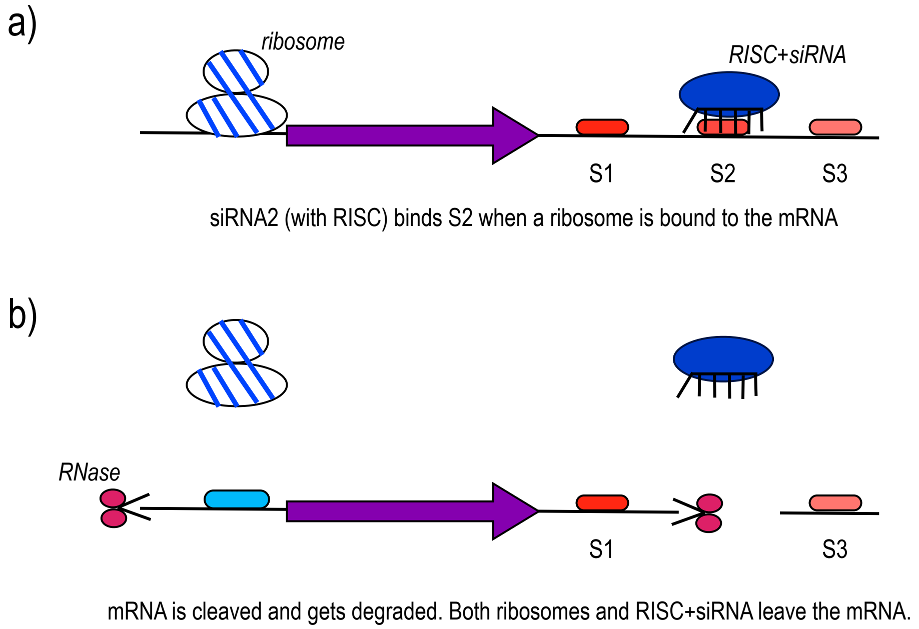

Figure 3.3: **Translation regulation via mRNA cleavage.** a) Small interfering RNAs bind their mRNA binding sites also when transcription is occurring. b) siRNAs cleave the mRNA and RNase molecules can degrade it fast. Under these circumstances, ribosomes abort transcription and leave the mRNA.

Chapter 4

Eukaryotic gene circuits: an example

If not explicitly indicated, references for parameter values are the same as in the previous chapter. Note that fluxes with the superscript "b" are *balance* fluxes that arise when molecules are exchanged between a part and a pool.

4.1 The constitutive p0 promoter

This promoter contains only the RNA polymerase binding site, *polsite*.

Species

$polsite$	RNA polymerase binding site
Pol^{free}	from the RNA polymerase pool
Pol^{cl}	fictitious species, converted into $PoPS^{out}$

Reactions and fluxes

$$Pol^{free} + polsite^{free} \overset{(k_1,k_{-1})}{\rightleftharpoons} polsite^{taken} \xrightarrow{k_2} polsite^{free} + Pol^{cl} \tag{4.1}$$

$$PoPS^{out} = k_2 polsite^{taken} \tag{4.2}$$

$$PoPS^b = k_1 polsite^{free} Pol^{free} - k_{-1} polsite^{taken} \tag{4.3}$$

BNGL molecule types

pol_cl()
pol_free
p(pol_site~free~taken)

BNGL rules

pol_free() + p(pol_site ~ free) < − > p(pol_site ~ taken) k1,k_1
p(pol_site ~ taken) − > p(pol_site ~ free) + pol_cl() k2

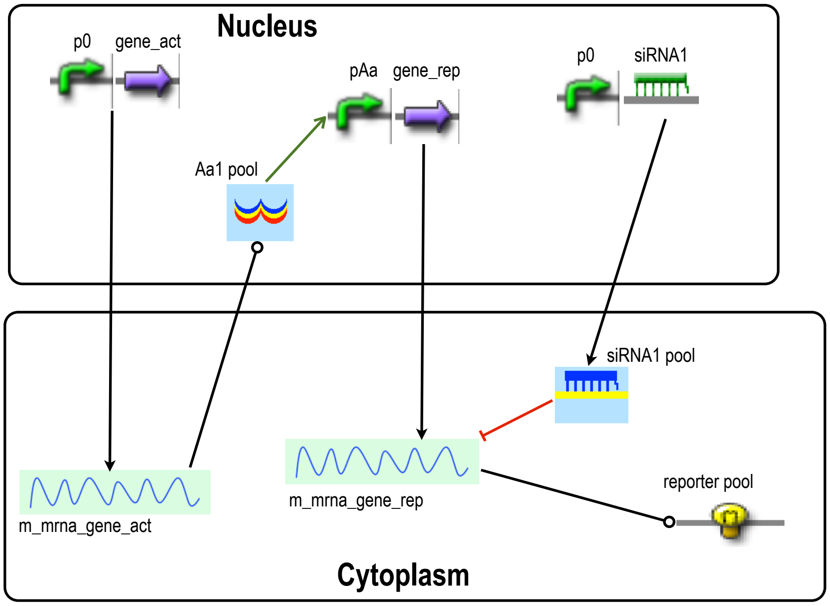

Figure 4.1: **Scheme of the circuit.** As shown in the main text, this is the simple circuit we chose in order to give an explanation of our model for eukaryotic parts and pools.

Parameters' values

Parameters	Values	
$polsite^{free}$	$4.4 \cdot 10^{-12}\, M$	(1 plasmid)
k_1	$10^5\, M^{-1} s^{-1}$	
k_{-1}	$1\, s^{-1}$	
k_2	$0.5\, s^{-1}$	

4.2 The regulated pAa promoter

Together with the RNA polymerase binding site *polsite*, this promoter contains two operators for a unique activator A_1^a: $Oa1_1$ and $Oa1_2$. Operators can be in two states: f, free or t, taken by A_1^a. Since A_1^a molecules bind cooperatively, $Oa1_2$ has a stronger affinity towards A_1^a.

Species

Pol^{free}	from the RNA polymerase pool
A_1^a	from the activator pool
Pol^{cl}	fictitious species, converted into $PoPS^{out}$
Pol^{cl}_{lk}	fictitious species, converted into $PoPS^{out}_{lk}$
$Oa1_1^f Oa1_2^f polsite^{free}$	
$Oa1_1^t Oa1_2^f polsite^{free}$	weak bond
$Oa1_1^f Oa1_2^t polsite^{free}$	strong bond
$Oa1_1^t Oa1_2^t polsite^{free}$	strong bond, RNA polymerase can bind the promoter
$Oa1_1^t Oa1_2^f polsite^{taken}$	this configuration arise because of A_1^a degradation on $Oa1_2$
$Oa1_1^t Oa1_2^t polsite^{taken}$	

Reactions and fluxes

$$A_1^a + Oa1_1^f Oa1_2^f polsite^{free} \overset{(\alpha_{1s},\beta_{1s})}{\rightleftharpoons} Oa1_1^f Oa1_2^t polsite^{free} \tag{4.4}$$

$$A_1^a + Oa1_1^t Oa1_2^f polsite^{free} \overset{(\alpha_{1s},\beta_{1s})}{\rightleftharpoons} Oa1_1^t Oa1_2^t polsite^{free} \tag{4.5}$$

$$A_1^a + Oa1_1^f Oa1_2^f polsite^{free} \overset{(\alpha_{1w},\beta_{1w})}{\rightleftharpoons} Oa1_1^t Oa1_2^f polsite^{free} \tag{4.6}$$

$$A_1^a + Oa1_1^f Oa1_2^t polsite^{free} \overset{(\alpha_{1c},\beta_{1c})}{\rightleftharpoons} Oa1_1^t Oa1_2^t polsite^{free} \tag{4.7}$$

$$Pol^{free} + Oa1_1^t Oa1_2^t polsite^{free} \overset{(k_1,k_{-1})}{\rightleftharpoons} Oa1_1^t Oa1_2^t polsite^{taken} \xrightarrow{k_2} Oa1_1^t Oa1_2^t polsite^{free} + Pol^{cl} \tag{4.8}$$

$$Oa1_1^t Oa1_2^t polsite^{free} \xrightarrow{k_{d1}} Oa1_1^f Oa1_2^t polsite^{free} \tag{4.9}$$

$$Oa1_1^t Oa1_2^t polsite^{free} \xrightarrow{k_{d1}} Oa1_1^t Oa1_2^f polsite^{free} \tag{4.10}$$

$$Oa1_1^t Oa1_2^f polsite^{free} \xrightarrow{k_{d1}} Oa1_1^f Oa1_2^f polsite^{free} \tag{4.11}$$

$$Oa1_1^f Oa1_2^t polsite^{free} \xrightarrow{k_{d1}} Oa1_1^f Oa1_2^f polsite^{free} \tag{4.12}$$

$$Oa1_1^t Oa1_2^t polsite^{taken} \xrightarrow{k_{d1}} Oa1_1^t Oa1_2^f polsite^{taken} \tag{4.13}$$

$$Oa1_1^t Oa1_2^t polsite^{taken} \xrightarrow{k_{d1}} Oa1_1^f Oa1_2^t polsite^{free} + Pol^{free} \tag{4.14}$$

$$PoPS^b = k_1 Oa1_1^t Oa1_2^t polsite^{free} - k_{-1} Oa1_1^t Oa1_2^t polsite^{taken} \tag{4.15}$$

$$PoPS^{out} = k_2 Oa1_1^t Oa1_2^t polsite^{taken} \tag{4.16}$$

$$PoPS^{lk} = k_2^{lk} (Oa1_1^f Oa1_2^f polsite^{free} + Oa1_1^f Oa1_2^t polsite^{free}) \tag{4.17}$$

$$\begin{aligned} FaPS^b = \; & + \; \alpha_{1s} A_1^a (Oa1_1^f Oa1_2^f polsite^{free} + Oa1_1^t Oa1_2^f polsite^{free}) + \\ & - \; \beta_{1s} (Oa1_1^f Oa1_2^t polsite^{free} + Oa1_1^t Oa1_2^t polsite^{free}) + \\ & + \; \alpha_{1w} A_1^a Oa1_1^f Oa1_2^f polsite^{free} - \beta_{1w} Oa1_1^t Oa1_2^f polsite^{free} + \\ & + \; \alpha_{1c} A_1^a Oa1_1^f Oa1_2^t polsite^{free} - \beta_{1c} Oa1_1^t Oa1_2^t polsite^{free} \end{aligned} \tag{4.18}$$

BNGL molecule types

pol_cl()
pol_cl_lk()
pol_free
Aa1
p(Oa1_1~free~taken_activator,Oa1_2~free~taken_activator,pol_site~free~taken)

BNGL rules

Aa1() + p(Oa1_2~free,pol_site~free) < − > p(Oa1_2~taken_activator,pol_site~free) alpha1_s_act,beta1_s_act
Aa1() + p(Oa1_1~free,Oa1_2~free,pol_site~free) < − >
p(Oa1_1~taken_activator,Oa1_2~free,pol_site~free) alpha1_w_act,beta1_w_act
Aa1() + p(Oa1_1~free,Oa1_2~taken_activator,pol_site~free) < − >
p(Oa1_1~taken_activator,Oa1_2~taken_activator,pol_site~free) alpha1_c_act,beta1_c_act
pol_free() + p(Oa1_1~taken_activator,Oa1_2~taken_activator,pol_site~free) < − >
p(Oa1_1~taken_activator,Oa1_2~taken_activator,pol_site~taken) k1,k_1
p(Oa1_1~taken_activator,pol_site~free) − > p(Oa1_1~free,pol_site~free) k_d1_act

p(Oa1_2~taken_activator,pol_site~free) − > p(Oa1_2~free,pol_site~free) k_d1_act
p(Oa1_1~taken_activator,pol_site~taken) − > p(Oa1_1~free,pol_site~free) + pol_free() k_d1_act
p(Oa1_2~taken_activator,pol_site~taken) − > p(Oa1_2~free,pol_site~taken) k_d1_act
p(Oa1_1~taken_activator,pol_site~taken) − > p(Oa1_1~taken_activator,pol_site~free) + pol_cl() k2
p(Oa1_1~free,pol_site~free) − > p(Oa1_1~free,pol_site~free) + pol_cl_lk() k2_lk

Parameters' values

α and β values have been chosen to mimic only partial cooperativity ([5]).

Parameters	Values	
$Oa1_1^f Oa1_2^f polsite^{free}$	$4.4 \cdot 10^{-12}\,M$	(1 plasmid)
k_1	$10^5\,M^{-1}s^{-1}$	
k_{-1}	$1\,s^{-1}$	
k_2	$0.5\,s^{-1}$	
k_2^{lk}	$0.5\,s^{-1}$	
α_{1s}	$10^9\,M^{-1}s^{-1}$	[5]
β_{1s}	$9\,s^{-1}$	[5]
α_{1w}	$10^9\,M^{-1}s^{-1}$	[5]
β_{1w}	$224\,s^{-1}$	[5]
α_{1c}	$10^9\,M^{-1}s^{-1}$	[5]
β_{1c}	$9\,s^{-1}$	[5]
k_{d1}	$2.8 \cdot 10^{-5}\,s^{-1}$	

4.3 Coding region for proteins: gene_aa1 and gene_rep

Species

Y^{free}	from the spliceosome pool
Pol^{el}	fictitious species, converted into $PoPS^{out}$
$[PolA]$	RNA polymerase bound to the DNA before starting elongation
u_{mrna}	unspliced mRNA
$[Yu_{mrna}]$	complex: spliceosome bound to u_{mrna}
n_{mrna}	nuclear mRNA as a splicing product
m_{mrna}	mature mRNA, sent to the corrresponding m_mrna pool in the cytoplasm as $RNAPS^{out}$

Reactions and fluxes

$$PoPS^{in} \Longrightarrow [PoA] \tag{4.19}$$

$$[PolA] \xrightarrow{k_{el}} Pol^{el} + u_{mrna} \tag{4.20}$$

$$Y^{free} + u_{mrna} \overset{(k_{1y},k_{-1y})}{\rightleftharpoons} [Yu_{mrna}] \xrightarrow{k_{2y}} Y^{free} + n_{mrna} \tag{4.21}$$

$$n_{mrna} \xrightarrow{k_m} m_{mrna} \tag{4.22}$$

$$n_{mrna} \xrightarrow{k_d} \tag{4.23}$$

$$u_{mrna} \xrightarrow{k_d} \tag{4.24}$$

$$[Yu_{mrna}] \xrightarrow{k_d} Y^{free} \tag{4.25}$$

$$RNAPS^{out} = k_m n_{mrna} \tag{4.26}$$

$$PoPS^{out} = k_{el}[PolA] \tag{4.27}$$

$$YPS^b = k_{1y}Y^{free}u_{mrna} - (k_{-1y} + k_{2y} + k_d)[Yu_{mrna}] \tag{4.28}$$

Parameters' values

k_d is specified into the terminator connected to the coding region.

Parameters	Values	
k_{1y}	$1.5 \cdot 10^5\, M^{-1}s^{-1}$	
k_{-1y}	$0.0017\, s^{-1}$	
k_{2y}	$0.033\, s^{-1}$	
k_m	$5.5 \cdot 10^{-4}\, s^{-1}$	
k_d	$1.9 \cdot 10^{-5}\, s^{-1}$	
v_{pol}	$23.3\, nt/s$	to calculate k_{el}

4.4 Coding region for siRNAs: siRNA1

Species

D^{free}	from the Dicer pool
Pol^{el}	fictitious species, converted into $PoPS^{out}$
$[PolA]$	RNA polymerase bound to the DNA before starting elongation
ds_{rna}	double stranded RNA
$[Dds_{rna}]$	complex: Dicer bound to ds_{rna}
n_{sirna}	nuclear siRNA
$siRNA$	mature siRNA, sent to the siRNA1 pool in the cytoplasm as $RNAPS^{out}$

Reactions and fluxes

$$PoPS^{in} \Longrightarrow [PoA] \tag{4.29}$$

$$[PolA] \xrightarrow{k_{el}} Pol^{el} + ds_{rna} \tag{4.30}$$

$$D^{free} + ds_{rna} \overset{(k_{1d},k_{-1d})}{\rightleftharpoons} [Dds_{rna}] \xrightarrow{k_{2d}} D^{free} + n_{sirna} \tag{4.31}$$

$$n_{sirna} \xrightarrow{k_m} siRNA \tag{4.32}$$

$$n_{sirna} \xrightarrow{k_d} \tag{4.33}$$

$$ds_{rna} \xrightarrow{k_d} \tag{4.34}$$

$$[Dds_{rna}] \xrightarrow{k_d} D^{free} \tag{4.35}$$

$$RNAPS^{out} = k_m n_{sirna} \tag{4.36}$$

$$PoPS^{out} = k_{el}[PolA] \tag{4.37}$$

$$DPS^b = k_{1d}D^{free}ds_{rna} - (k_{-1d} + k_{2d} + k_d)[Dds_{rna}] \tag{4.38}$$

Parameters' values

k_d is specified into the terminator connected to the siRNA coding region.

Parameters	Values	
k_{1d}	$1.5 \cdot 10^5\, M^{-1}s^{-1}$	
k_{-1d}	$0.0017\, s^{-1}$	
k_{2d}	$0.033\, s^{-1}$	
k_m	$5.5 \cdot 10^{-4}\, s^{-1}$	
k_d	$1.9 \cdot 10^{-5}\, s^{-1}$	
v_{pol}	$23.3\, nt/s$	to calculate k_{el}

4.5 Terminators

Species

$[PolT]$	RNA polymerase bound to the terminator
Pol^{free}	sent as $PoPS^{out}$ to the RNA polymerase pool

Reactions and fluxes

$$PoPS^{in} \Longrightarrow [PoT] \tag{4.39}$$

$$[PolTA] \xrightarrow{\zeta} Pol^{free} \tag{4.40}$$

$$PoPS^{out} = \zeta[PolT] \tag{4.41}$$

Parameters' values

Parameters	Values
ζ	$31.25\, s^{-1}$

4.6 The activator pool (Aa1 pool)

Species

A_1^{am}	monomeric A_1^a
A_1^a	dimeric A_1^a, this binds the DNA

Reactions and fluxes

$$FaPS^{in} \Longrightarrow A_1^{am} \tag{4.42}$$

$$2A_1^{am} \overset{(\delta,\epsilon)}{\rightleftharpoons} A_1^a \tag{4.43}$$

$$A_1^{am} \xrightarrow{k_{d1}} \tag{4.44}$$

$$A_1^a \xrightarrow{k_{d1}} \tag{4.45}$$

Parameters' values

Parameters	Values
δ	$10^9\, M^{-1}s^{-1}$
ϵ	$10\, s^{-1}$
k_{d1}	$2.8 \cdot 10^{-5}\, s^{-1}$

4.7 m_mrna_gene_act

The mRNA that encodes the A_1^a activator is not regulated and contains only a binding site b for the ribosomes.

Species

$ribsite$	ribosome binding site
rib^{free}	from the ribosome pool
$ribSTART$	ribosome bound to the mRNA at the START codon, before starting elongation
$ribSTOP$	ribosome bound to the mRNA at the STOP codon, before leaving the mRNA pool
$protein_c$	proteins in cytoplasm, before being imported into the nucleus
$protein$	sent as $FaPS^{out}$ to the A_1^a pool

Reactions and fluxes

$$rib^{free} + ribsite^{free} \overset{(k_{1r},k_{-1r})}{\rightleftharpoons} ribsite^{taken} \xrightarrow{k_{2r}} ribsite^{free} + ribSTART \tag{4.46}$$

$$ribSTART \xrightarrow{k^r_{el}} ribSTOP \tag{4.47}$$

$$ribSTOP \xrightarrow{\zeta^r} rib^{free} + protein_c \tag{4.48}$$

$$protein_c \xrightarrow{k_{tr}} protein \tag{4.49}$$

$$protein_c \xrightarrow{k_{dp}} \tag{4.50}$$

$$ribsite^{free} \xrightarrow{k_d} \tag{4.51}$$

$$ribsite^{taken} \xrightarrow{k_d} rib^{free} \tag{4.52}$$

$$FaPS^{out} = k_{tr} protein_c \tag{4.53}$$

$$RiPS^b = k_{1r} ribsite^{free} rib^{free} - k_{-1r} ribsite^{taken} \tag{4.54}$$

$$RiPS^{out} = \zeta^r ribSTOP + k_d ribsite^{taken} \tag{4.55}$$

BNGL molecule types

Notice: Trash() is required by BioNetGen to handle degradation.
b(rib_site~free~taken)
rib_free()
ribSTART()
ribSTOP()
protein_c()
protein()
Trash()

BNGL rules

rib_free() + b(rib_site~free) < − > b(rib_site~taken) k1r,k_1r
b(rib_site~taken) − > b(rib_site~free) + ribSTART() k2r
ribSTART() − > ribSTOP() k_el_r
ribSTOP() − > rib_free() + protein_c() zeta_r
protein_c() − > protein() k_tr
protein_c() − > Trash() k_dp
b(rib_site~free) − > Trash() k_d
b(rib_site~taken) − > Trash() + rib_free() k_d

Parameters' values

Note that k_d is passed by the part gene_act

Parameters	Values	
k_{1r}	$10^6\, M^{-1}s^{-1}$	
k_{-1}	$0.01\, s^{-1}$	
k_{2r}	$0.02\, s^{-1}$	
v_{rib}	$24.0\, nt/s$	to calculate k^r_{el}
ζ^r	$0.5\, s^{-1}$	
k_{dp}	$2.8 \cdot 10^{-5}\, s^{-1}$	Corresponds to k_{d1} i.e. A^a_1 decay rate
k_{tr}	$8.3.5 \cdot 10^{-3}\, s^{-1}$	

4.8 m_mrna_gene_rep

The mRNA that encodes the reporter protein is regulated by siRNA1 that can bind two sites $S1_1$ and $S1_2$. siRNA binding sites have two possible states: unbound (”on”) and bound (”off”). Ribosomes bind the mRNA only when both siRNA1 binding sites are ”on”. Moreover, when siRNA1 binds one of the two sites, the mRNA gets cleaved and it is degraded quickly. As in our model for the bacterial RBS, $PoPS^{in}_{lk}$ increases directly the concentration of ”free” mRNA i.e. not bound both by ribosomes and siRNA1.

Species

$S_1^{on}S_2^{on}ribsite^{free}$	
$S_1^{off}S_2^{on}ribsite^{free}$	to be cleaved
$S_1^{on}S_2^{off}ribsite^{free}$	to be cleaved
$S_1^{off}S_2^{on}ribsite^{taken}$	to be cleaved
$S_1^{on}S_2^{off}ribsite^{taken}$	to be cleaved
$PoPS^{in}_{lk}$	from the pAa promoter
rib^{free}	from the ribosome pool
$s1$	siRNA1-RISC complex, from the siRNA1 pool
$ribSTART$	ribosome bound to the mRNA at the START codon, before starting elongation
$ribSTOP$	ribosome bound to the mRNA at the STOP codon, before leaving the mRNA pool
$protein_c$	proteins in cytoplasm, before being imported into the nucleus
$protein$	sent as $FaPS^{out}$ to the A_1^a pool

Reactions and fluxes

$$PoPS^{in}_{lk} \Longrightarrow S_1^{on}S_2^{on}ribsite^{free} \tag{4.56}$$

$$s1 + S_1^{on}S_2^{on}ribsite^{free} \overset{(\theta_{s1},\xi_{s1})}{\rightleftharpoons} S_1^{off}S_2^{on}ribsite^{free} \tag{4.57}$$

$$s1 + S_1^{on}S_2^{on}ribsite^{free} \overset{(\theta_{s1},\xi_{s1})}{\rightleftharpoons} S_1^{on}S_2^{off}ribsite^{free} \tag{4.58}$$

$$s1 + S_1^{on}S_2^{on}ribsite^{taken} \overset{(\theta_{s1},\xi_{s1})}{\rightleftharpoons} S_1^{off}S_2^{on}ribsite^{taken} \tag{4.59}$$

$$s1 + S_1^{on}S_2^{on}ribsite^{taken} \overset{(\theta_{s1},\xi_{s1})}{\rightleftharpoons} S_1^{on}S_2^{off}ribsite^{taken} \tag{4.60}$$

$$rib^{free} + S_1^{on}S_2^{on}ribsite^{free} \overset{(k_{1r},k_{-1r})}{\rightleftharpoons} S_1^{on}S_2^{on}ribsite^{taken} \xrightarrow{k_{2r}} S_1^{on}S_2^{on}ribsite^{free} + ribSTART \tag{4.61}$$

$$ribSTART \xrightarrow{k^r_{el}} ribSTOP \tag{4.62}$$

$$ribSTOP \xrightarrow{\zeta^r} rib^{free} + protein_c \tag{4.63}$$

$$protein_c \xrightarrow{k_{tr}} protein \tag{4.64}$$

$$protein_c \xrightarrow{k_{dp}} \tag{4.65}$$

$$S_1^{on}S_2^{on}ribsite^{free} \xrightarrow{k_d} \tag{4.66}$$

$$S_1^{off}S_2^{on}ribsite^{free} \xrightarrow{k_{fd}} s1 \tag{4.67}$$

$$S_1^{on}S_2^{off}ribsite^{free} \xrightarrow{k_{fd}} s1 \tag{4.68}$$

$$S_1^{on}S_2^{on}ribsite^{taken} \xrightarrow{k_d} rib^{free} \tag{4.69}$$

$$S_1^{off}S_2^{on}ribsite^{taken} \xrightarrow{k_{fd}} s1 + rib^{free} \tag{4.70}$$

$$S_1^{on}S_2^{off}ribsite^{taken} \xrightarrow{k_{fd}} s1 + rib^{free} \tag{4.71}$$

$$RiPS^b = k_{1r}S_1^{on}S_2^{on}ribsite^{free}rib^{free} - S_1^{on}S_2^{on}k_{-1r}ribsite^{taken} \tag{4.72}$$

$$RiPS^{out} = \zeta^r ribSTOP + k_d S_1^{on}S_2^{on}ribsite^{taken} + k_{df}(S_1^{off}S_2^{on}ribsite^{taken} + S_1^{on}S_2^{off}ribsite^{taken}) \tag{4.73}$$

$$\begin{aligned} RNAPS^b &= 2\,\theta_{s1}s1(S_1^{on}S_2^{on}ribsite^{free} + S_1^{on}S_2^{on}ribsite^{taken}) + \\ &- \xi_{s1}(S_1^{on}S_2^{off}ribsite^{free} + S_1^{off}S_2^{on}ribsite^{free} + S_1^{on}S_2^{off}ribsute^{taken} + S_1^{off}S_2^{on}ribsite^{taken}) + \\ &+ k_{fd}(S_1^{off}S_2^{on}ribsite^{free} + S_1^{on}S_2^{off}ribsite^{free} + S_1^{off}S_2^{on}ribsite^{taken} + S_1^{on}S_2^{off}ribsite^{taken}) \end{aligned} \tag{4.74}$$

BNGL molecule types

Notice: Trash() is required by BioNetGen to handle degradation.

b(S1_1~on~off,S1_2~on~off,rib_site~free~taken)
rib_free()
s1()
ribSTART()
ribSTOP()
pops_in_lk()
protein_c()
protein()
Trash()

BNGL rules

pops_in_lk() − > b(S1_1~on,S1_2~on,rib_site~free) pops_in_lk
s1() + b(S1_1~on,S1_2~on) < − > b(S1_1~off,S1_2~on) theta_s1,csi_s1
s1() + b(S1_2~on,S1_1~on) < − > b(S1_2~off,S1_1~on) theta_s1,csi_s1
rib_free() + b(S1_1~on,S1_2~on,rib_site~free) < − > b(S1_1~on,S1_2~on,rib_site~taken) k1r,k_1r
b(S1_1~on,S1_2~on,rib_site~taken) − > b(S1_1~on,S1_2~on,rib_site~free) + ribSTART() k2r
ribSTART() − > ribSTOP() k_el_r
ribSTOP() − > rib_free() + protein_c() zeta_r
protein_c() − > protein() k_tr
protein_c() − > Trash() k_dp
b(S1_1~on,S1_2~on,rib_site~free) − > Trash() k_d
b(S1_1~off,S1_2~on,rib_site~free) − > Trash() + s1() k_fd
b(S1_2~off,S1_1~on,rib_site~free) − > Trash() + s1() k_fd
b(S1_1~on,S1_2~on,rib_site~taken) − > Trash() + rib_free() k_d
b(S1_1~off,S1_2~on,rib_site~taken) − > Trash() + rib_free() + s1() k_fd
b(S1_2~off,S1_1~on,rib_site~taken) − > Trash() + rib_free() + s1() k_fd

Parameters' values

Note that k_d is passed by the part gene_rep.

Parameters	Values	
k_{1r}	$10^5\,M^{-1}s^{-1}$	
k_{-1}	$1\,s^{-1}$	
k_{2r}	$0.02\,s^{-1}$	
v_{rib}	$24.0\,nt/s$	to calculate k^r_{el}
ζ^r	$0.5\,s^{-1}$	
k_{dp}	$2.8 \cdot 10^{-5}\,s^{-1}$	
k_{tr}	$8.3.5 \cdot 10^{-3}\,s^{-1}$	
k_{fd}	$2 \cdot 10^{-3}\,s^{-1}$	
θ_{s1}	$10^7\,M^{-1}s^{-1}$	
ξ_{s1}	$0.01\,s^{-1}$	

4.9 The siRNA pool (siRNA1 pool)

Species

$siRNA_1$	
$RISC^{free}$	from the corresponding pool
$[risi]$	siRNA1 bound to RISC

Reactions and fluxes

$$RNAPS^{in} \Longrightarrow siRNA1 \tag{4.75}$$

$$RISC^{free} + siRNA1 \overset{(k_{1risc},k_{-1risc})}{\rightleftharpoons} [risi] \tag{4.76}$$

$$[risi] \xrightarrow{k_d} RISC^{free} \tag{4.77}$$

$$RISCPS^b = k_{1risc}RISC^{free}siRNA1 - (k_{-1risc} + k_d)[risi] \tag{4.78}$$

Parameters' values

Note that k_d is passed by the part siRNA1.

Parameters	Values
k_{1risc}	$3.0 \cdot 10^7\,M^{-1}s^{-1}$
k_{-1risc}	$0.017\,s^{-1}$

4.10 The reporter protein pool

Species

rep

Reactions and fluxes

$$FaPS^{in} \Longrightarrow rep \tag{4.79}$$

$$rep \xrightarrow{k_d} \tag{4.80}$$

Parameters' values

Parameters	Values	
k_d	$2.8 \cdot 10^{-5}\,s^{-1}$	passed by its coding region part

4.11 Other pools

RNA polymerase, ribosome, spliceosome, Dicer and RISC pools do not contain any reaction. The amount of free molecules of each of these species is constantly updated according to the following differential equation

$$\frac{d\,molecules^{free}}{dt} = flux^{in} - flux^{out} = flux^{b} \quad . \tag{4.81}$$

4.12 Simulations

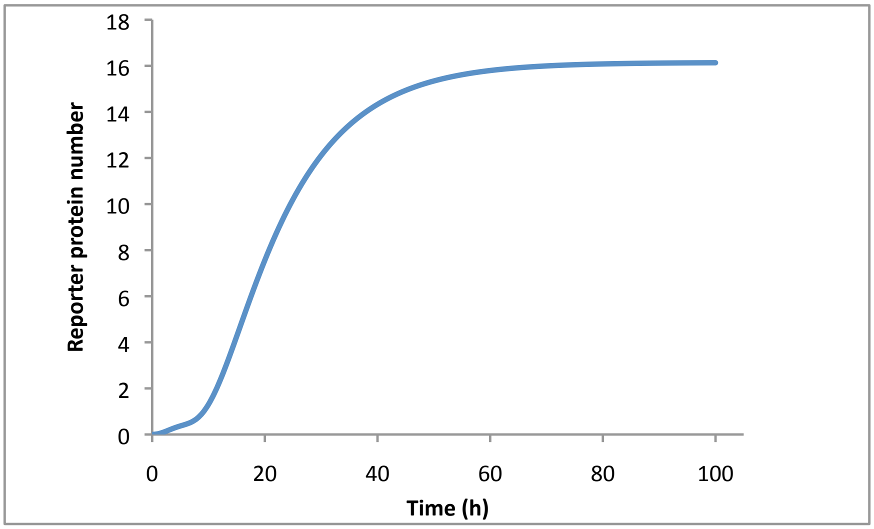

Figure 4.2: **Circuit simulation.** As expected, the repressive action of siRNA1 limits the reporter protein expression. Only ~ 16 proteins are present in the cell after 100 hours.

Chapter 5

Logic evaluator simulations

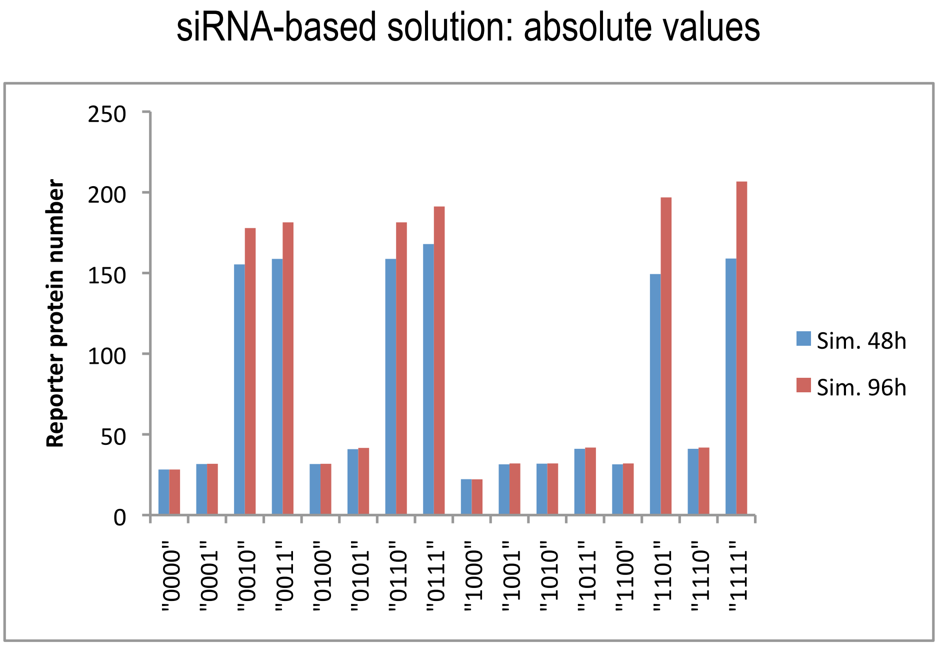

Figure 5.1: **Logic evaluator: absolute values.** RNAi-based circuit. A 96-hour simulation seems to approach the circuit steady state better. However, the signal separation does not increase dramatically with respect to the 48-hour results.

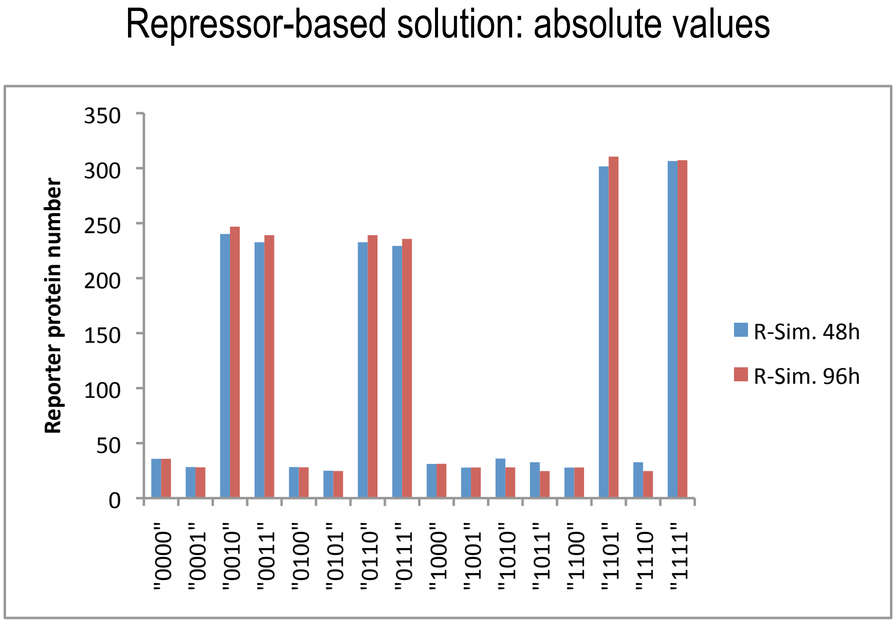

Figure 5.2: **Logic evaluator: absolute values.** Transcription repression-based. A longer simulation has no apparent effect on the signal separation.

Bibliography

[1] B. Albers, A. Johnson, J. Lewis, M. Raff, K. Roberts, and P. Walter. *Molecular Biology of the Cell*. Garland Science, 5 edition, 2008.

[2] D. W. Bartlett and M. E. Davis. Insights into the kinetics of sirna-mediated gene silencing from live-cell and live-animal bioluminescent imaging. *Nucleic Acids Res*, 34(1):322–333, 2006.

[3] S. B.-T. de Leon and E. H. Davidson. Modeling the dynamics of transcriptional gene regulatory networks for animal development. *Dev Biol*, 325(2):317–328, Jan 2009.

[4] E. Eden, N. Geva-Zatorsky, I. Issaeva, A. Cohen, E. Dekel, T. Danon, L. Cohen, A. Mayo, and U. Alon. Proteome half-life dynamics in living human cells. *Science*, 331(6018):764–768, Feb 2011.

[5] M. B. Elowitz and S. Leibler. A synthetic oscillatory network of transcriptional regulators. *Nature*, 403(6767):335–338, Jan 2000.

[6] A. Fujioka, K. Terai, R. E. Itoh, K. Aoki, T. Nakamura, S. Kuroda, E. Nishida, and M. Matsuda. Dynamics of the ras/erk mapk cascade as monitored by fluorescent probes. *J Biol Chem*, 281(13):8917–8926, Mar 2006.

[7] M. A. Marchisio and J. Stelling. Computational design of synthetic gene circuits with composable parts. *Bioinformatics*, 24(17):1903–1910, Sep 2008.

[8] G. G. Maul and L. Deaven. Quantitative determination of nuclear pore complexes in cycling cells with differing dna content. *J Cell Biol*, 73(3):748–760, Jun 1977.

[9] B. O. Pallson. *Systems Biology: Properties of Reconstructed Networks*. Cambridge University Press, 2006.

[10] M. Piques, W. X. Schulze, M. Hhne, B. Usadel, Y. Gibon, J. Rohwer, and M. Stitt. Ribosome and transcript copy numbers, polysome occupancy and enzyme dynamics in arabidopsis. *Mol Syst Biol*, 5:314, 2009.

[11] D. S. Ucker and K. R. Yamamoto. Early events in the stimulation of mammary tumor virus rna synthesis by glucocorticoids. novel assays of transcription rates. *J Biol Chem*, 259(12):7416–7420, Jun 1984.
